# Genetic variation and population structure of Sudanese populations as indicated by 15 Identifiler sequence-tagged repeat (STR) loci

**DOI:** 10.1186/2041-2223-2-12

**Published:** 2011-05-04

**Authors:** Hiba MA Babiker, Carina M Schlebusch, Hisham Y Hassan, Mattias Jakobsson

**Affiliations:** 1 Department of Evolutionary Biology, Evolutionary Biology Centre, Uppsala University, Norbyvägen 18D, SE-752 36 Uppsala, Sweden; 2 Department of Evolutionary Genetics, Max-Planck Institute for Evolutionary Biology, August-Thienemann-Str. 2, D-24306 Plön, Germany; 3 University of Science and Technology, College of Medical Laboratory Sciences, Khartoum, Sudan

## Abstract

**Background:**

There is substantial ethnic, cultural and linguistic diversity among the people living in east Africa, Sudan and the Nile Valley. The region around the Nile Valley has a long history of succession of different groups, coupled with demographic and migration events, potentially leading to genetic structure among humans in the region.

**Result:**

We report the genotypes of the 15 Identifiler microsatellite markers for 498 individuals from 18 Sudanese populations representing different ethnic and linguistic groups. The combined power of exclusion (PE) was 0.9999981, and the combined match probability was 1 in 7.4 × 10^17^. The genotype data from the Sudanese populations was combined with previously published genotype data from Egypt, Somalia and the Karamoja population from Uganda. The Somali population was found to be genetically distinct from the other northeast African populations. Individuals from northern Sudan clustered together with those from Egypt, and individuals from southern Sudan clustered with those from the Karamoja population. The similarity of the Nubian and Egyptian populations suggest that migration, potentially bidirectional, occurred along the Nile river Valley, which is consistent with the historical evidence for long-term interactions between Egypt and Nubia.

**Conclusion:**

We show that despite the levels of population structure in Sudan, standard forensic summary statistics are robust tools for personal identification and parentage analysis in Sudan. Although some patterns of population structure can be revealed with 15 microsatellites, a much larger set of genetic markers is needed to detect fine-scale population structure in east Africa and the Nile Valley.

## Background

Sudan is located in northeastern Africa, with a total of 133 living languages listed by Ethnologue [1]. Local languages belong to three of the major African linguistic families proposed by Greenberg [2]: the Niger-Congo, Nilo-Saharan and Afro-Asiatic language families. The considerable ethnic and cultural diversity within Sudan make the study of existing genetic diversity of human populations an attractive effort. The Nile Valley has a long history of succession of different groups, coupled with demographic and migration events, which remain to be fully examined on a genetic level. These groups include people with an established history in the area, (for example, Nuba and Nilotic) and groups that migrated to the area in relatively recent times (for example, Hausa, Copt and Arab). Furthermore, population samples from Sudan are important for studies concerning human migration and the exodus from Africa 60,000 to 80,000 years ago, because the Nile Valley runs through Sudan, which is part of the traditionally favored model of the migratory route out of Africa for anatomically modern humans [3]. Previous genetic studies in Sudan have mainly focused on mitochondrial (mt)DNA, the Y chromosome [4-8], and a small number of autosomal markers [9-12]. Recently, Tishkoff *et al. *[13], conducted a large survey of 121 African populations using more than 800 microsatellites that included six populations from Sudan. Three of these populations were Nilotic populations, and one was a Nuba population, and these four populations speak Nilo-Saharan languages. The remaining two populations are Beja, who speak Afro-Asiatic languages. The study by Tishkoff *et al. *[13] showed that eastern Africa harbors substantial amounts of genetic diversity, only superseded by the amount of genetic diversity in southern Africa, but it is difficult to rank these regions, because of the very different sample density across Africa.

Since the introduction of a standardized set of forensic microsatellite markers [14], over 1000 populations across the world have been studied using these genetic markers [15]. The main results from these studies have been used to generate relevant reference data for a large number of populations, and to demonstrate that the set of microsatellite markers were sufficiently diverse to result in high PE (or low match probability) for particular populations. One of the more common commercial sequence-tagged repeat (STR) kits available for human identity testing is the AmpFlSTR^® ^Identifiler™ PCR Amplification Kit (Applied Biosystems, Foster City, CA, USA), which includes the 13 core STR loci from the FBI Combined DNA Index System (CODIS), and two additional markers commonly used for forensic investigations in Europe. The marker panel has also been used in population structure and admixture studies of humans [16-21], and knowledge about population structure has contributed to our understanding of human origins [22]. Admixture or ancestry analysis is also important in forensics; for instance, to pinpoint an appropriate reference population for a particular case from which to compute match and exclusion probabilities, or to potentially get an indication of the perpetrator's ethnicity [23]. However, recent studies investigating the informativeness for ancestry inference of the CODIS markers have suggested that these markers are less informative about ancestry than are many other marker sets of similar size [16,23,24].

In this study, we report the genotypes of 15 autosomal STR markers (the markers in the AmpFlSTR Identifiler PCR Amplification Kit) for different populations in Sudan, and compute commonly used forensic summary statistics. We infer population structure for the Sudanese population and for an expanded set of populations (compiled from previous studies) from Uganda [25], Egypt [26] and Somalia [27]. Lastly, we characterize the informativeness of the 15 forensic STR markers for assignment for the populations from northeast Africa and compare the result to another set of microsatellites.

## Results and discussion

**Figure 1:**
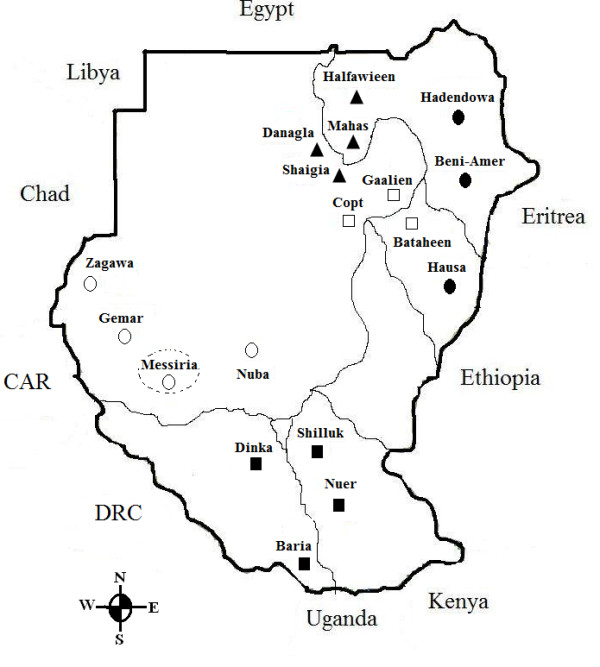
**Map of the Sudan representing the geographic locations of the 18 populations in the present study**. Geographic classification (used in the partitioning of variation analysis) of each population is also indicated on the map: northern Sudan (triangle), central Sudan (open square), eastern Sudan (filled circle), western Sudan (open circle) and southern Sudan (filled square). The dotted circle represents the suggested region occupied by the Messiria population.

The geographic locations of the sampled populations are indicated on a map of Sudan (Figure 1), and sample sizes for the populations are given in Table 1. For the population-genetics analyses, we also combined the genotype data from our Sudanese sample (454 individuals) with previously published genotype data from Uganda [25], Egypt [26] and Somalia [27].

**Table 1:** Sample sizes (*n*) and linguistic affiliations of the populations in the study

Ethnic group	Population	*n*	Linguistic family	Linguistic subgroup	Geographic group
Arab	Bataheen	29	Afro-Asiatic	Semetic	Central
Arab	Gaalien	57	Afro-Asiatic	Semetic	Central
Arab	Shaigia	17	Afro-Asiatic	Semetic	Northern
Arab	Messiria	8	Afro-Asiatic	Semetic	Western
Copt	Copt	31	Afro-Asiatic	Ancient Egyptian	Central
Hausa	Hausa	10	Afro-Asiatic	Chadic	Eastern
Beja	Beni-Amer	35	Afro-Asiatic	Cushitic	Eastern
Beja	Hadendowa	29	Afro-Asiatic	Cushitic	Eastern
Nubian	Danagla	40	Nilo-Saharan	Eastern Sudanic	Northern
Nubian	Mahas	31	Nilo-Saharan	Eastern Sudanic	Northern
Nubian	Halfawieen	24	Nilo-Saharan	Eastern Sudanic	Northern
Nilotic	Dinka	30	Nilo-Saharan	Eastern Sudanic	Southern
Nilotic	Nuer	19	Nilo-Saharan	Eastern Sudanic	Southern
Nilotic	Shilluk	19	Nilo-Saharan	Eastern Sudanic	Southern
Nilotic	Baria	6	Nilo-Saharan	Eastern Sudanic	Southern
Nuba	Nuba	34	Nilo-Saharan and Niger-Congo	Eastern Sudanic and Kordofanian	Western
Zagawa	Zagawa	29	Nilo-Saharan	Saharan	Western
Gemar	Gemar	6	Nilo-Saharan	Eastern Sudanic	Western

For the combination of the 18 Sudanese populations, allele frequencies for the 15 STR loci are shown in Table 2. Commonly used forensic summary statistics and deviations from Hardy-Weinberg equilibrium (HWE) for each locus are shown in Table 3. The D18S51 locus was found to have the greatest value of expected heterozygosity and it was also the locus with the lowest match probability, and the greatest powers of exclusion and discrimination (Table 3). The combined PE for the 15 loci was 99.99981%, and the combined match probability was 1.35 × 10^-18^. Five of the 15 loci (D13S317, D2S1338, D7S820, D8S1179, vWA; *P *= 0.021, 0.022, 0.015, 0.005 and 0.018, respectively, Fisher's exact test [28]) had deviation from HWE at the 5% level, suggesting a systematic, non-locus-specific deviation from HWE, potentially caused by population structure (see below). However, no individual locus deviated significantly from HWE after applying Bonferroni correction for 15 tests (adjusted *P *= 0.003).

**Table 2:** Allele frequencies for each of the 15 Identifiler STR loci found in the Sudanese sample.

Allele	CSF1PO	D13S317	D16S539	D18S51	D19S433	D21S11	D2S1338	D3S1358	D5S818	D7S820	D8S1179	FGA	TH01	TPOX	vWA
4													0.001		
6													0.228	0.006	
7	0.007								0.001	0.004	0.001		0.397	0.005	
8	0.048	0.069	0.039	0.002					0.084	0.228	0.001		0.091	0.390	
9	0.037	0.048	0.189	0.001	0.002				0.037	0.106	0.003		0.167	0.292	
9.3													0.083		
10	0.316	0.027	0.069	0.004	0.014				0.098	0.343	0.027		0.034	0.100	
10.2				0.001											
11	0.242	0.311	0.320	0.010	0.019				0.202	0.212	0.053			0.189	
11.2				0.001	0.004										
12	0.292	0.384	0.205	0.101	0.075			0.001	0.366	0.091	0.113			0.017	0.002
12.2					0.010										
13	0.048	0.128	0.147	0.087	0.225			0.003	0.203	0.015	0.241			0.001	0.003
13.2				0.002	0.055										
14	0.008	0.031	0.031	0.096	0.261			0.065	0.008		0.298				0.077
14.2				0.001	0.078										
15		0.003	0.001	0.111	0.092		0.001	0.305	0.001		0.188				0.156
15.2					0.082			0.001							
16				0.136	0.033		0.051	0.263			0.060				0.259
16.2				0.001	0.033			0.001							
17				0.140	0.008		0.139	0.271			0.010	0.008			0.245
17.2				0.002	0.007										
18				0.114	0.001		0.091	0.082			0.003	0.009			0.155
18.2					0.001							0.001			
19				0.078			0.209	0.005			0.001	0.047			0.075
19.2				0.001								0.001			
20				0.059			0.128	0.002				0.065			0.024
21				0.034			0.060					0.108			0.003
21.2												0.005			
22				0.013			0.128					0.183			0.001
22.2												0.002			
23				0.003			0.091					0.166			
24							0.056					0.143			
24.2						0.003						0.002			
25							0.035					0.091			
25.2						0.001									
26						0.002	0.009					0.039			
27						0.030	0.001					0.026			
28						0.123	0.001					0.063			
29						0.286						0.035			
30						0.226									
30.2						0.005						0.006			
31						0.082									
31.2						0.039									
32						0.017									
32.2						0.080									
33						0.007									
33.2						0.034									
34						0.009									
34.2						0.005									
35						0.026									
36						0.017									
37						0.005									
38						0.002									

See table 3 for exact sample size (n) for each locus

**Table 3:** Forensic summary statistics, observed and expected heterozygosity, deviation from Hardy-Weinberg-equilibrium (HWE), minimum allele frequency cut-off and sample size for each locus (n)

Locus	CSF1PO	D13S317	D16S539	D18S51	D19S433	D21S11	D2S1338	D3S1358	D5S818	D7S820	D8S1179	FGA	TH01	TPOX	vWA
n	485	489	491	485	491	491	489	490	490	485	491	486	490	491	491
MP^a^	0.103	0.114	0.073	0.021	0.043	0.047	0.028	0.106	0.090	0.093	0.069	0.026	0.100	0.126	0.066
PD^b^	0.897	0.886	0.927	0.979	0.957	0.953	0.972	0.894	0.91	0.907	0.931	0.974	0.9	0.874	0.934
PE^c^	0.504	0.409	0.552	0.785	0.665	0.611	0.68	0.533	0.519	0.469	0.503	0.748	0.431	0.406	0.681
PIC^d^	0.708	0.689	0.762	0.889	0.833	0.816	0.865	0.711	0.732	0.729	0.769	0.874	0.711	0.67	0.79
H_obs_^e^	0.746	0.687	0.774	0.895	0.833	0.804	0.843	0.763	0.755	0.726	0.745	0.877	0.702	0.684	0.841
H_exp_^f^	0.751	0.731	0.792	0.899	0.850	0.834	0.878	0.754	0.767	0.766	0.798	0.886	0.747	0.718	0.813
P^g^	0.955	0.021	0.335	0.357	0.195	0.211	0.022	0.335	0.258	0.015	0.005	0.749	0.311	0.400	0.018
MAF^h^	0.0052	0.0051	0.005	0.0052	0.005	0.005	0.0051	0.0051	0.0051	0.0052	0.005	0.0052	0.0051	0.005	0.005

^a^Matching probability.

^b^Power of discrimination.

^c^Power of exclusion.

^d^Polymorphism information content.

^e^Observed heterozygosity.

^f^Expected heterozygosity.

^g^HWE, Fisher's exact test *P*-value executed with 100,000 steps in the Markov chain and 1,000 dememorization steps.

^h^Minimum allele frequency cut-off (5/(2N)).

### Informativeness for assignment

Rosenberg *et al. *[29] developed a statistic, the informativeness for assignment (*Ι*_n_), which describes the information content of a particular genetic marker for ancestry inference (it ranges from zero (no information) to the natural logarithm of the number of populations (maximum information)). We computed *Ι*_n _values for the 15 Identifiler STRs (all tetranucleotide microsatellites) from the present study. The mean, across markers, (and standard deviation) *Ι*_n _was 0.167 (0.070) for the Sudanese populations, and 0.154 (0.066) for the Sudanese populations and the populations from Egypt, Somalia and Uganda (the Karamoja). To determine the relative informativeness of the 15 Identifier microsatellites, we computed *I*_n _for 377 microsatellites (of which 274 were tetranucleotide microsatellites) for two groups of African populations from Rosenberg *et al. *[29]; six sub-Saharan African populations (Kenyan Bantu speakers, Mandenka, Yoruba, San, Mbuti Pygmy, Biaka Pygmy) from the Human Genome Diversity Panel (HGDP); the 'HGDP sub-Saharan group'; and a subgroup of three populations from the HGDP, who all speak Niger-Congo languages (Kenyan Bantu speakers, Mandenka, Yoruba), termed the 'HGDP Niger-Congo group'. Based on the 377 microsatellites from Rosenberg *et al. *[29], the mean *I*_n _(across markers) was 0.234 (0.098) for the HGDP sub-Saharan group and 0.106 (0.054) for the HGDP Niger-Congo group, which was a significant difference (*P *< 0.001, Wilcoxon signed-rank test). The *I*_n _statistic depends on both marker information about ancestry and the level of differentiation between the investigated populations. Because the same markers were used for both the HGDP sub-Saharan group and the HGDP Niger-Congo group, the difference in *I*_n _between the two groups was due to the greater level of differentiation between populations in the HGDP sub-Saharan group. The *I*_n _values for the Sudanese populations and the larger group of northeast African populations (from Sudan, Egypt, Somalia and Uganda) were found to lie between the values for the HGDP Niger-Congo group and the HGDP sub-Saharan group. This result is not surprising, considering that the sampled populations from Sudan belonged to different linguistic groups, although the groups are not as differentiated as the Pygmy, the San and the Niger-Congo-speaking populations in the HGDP sub-Saharan group. We concluded that the CODIS STRs contain information on population structure in the same range as many other microsatellites. However, it is possible to select more informative STR loci for population structure inference; for example, the top 15 most informative loci of the 377 microsatellites [29] (for the HGDP sub-Saharan group and for the HGDP Niger-Congo group) had mean *I*_n _values of 0.489 (0.062) and 0.249 (0.041) respectively. This observation can be explained by the fact that the Identifiler STRs have been selected to have high levels of variation, because of the potentially high mutation rates of these STRs, in order to be powerful tools for identifying individuals, but the high level of variation also makes it challenging to separate alleles that are identical by state from those identical by descent [15].

### Population diversity and sub-structure in Sudanese and neighboring populations

**Figure 2:**
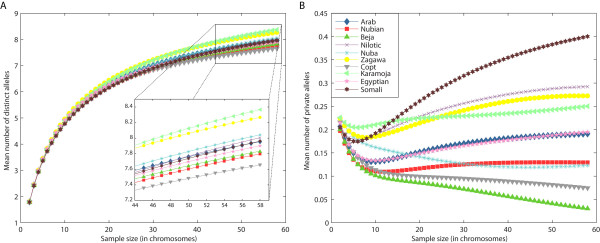
**The mean number of **(A) **distinct and **(B) **private alleles as functions of the sample size *g *(in chromosomes) for Sudanese populations and neighboring populations**.

The greatest number of distinct alleles [30], considering a sample size of 58 chromosomes (29 individuals) from each of the Sudanese populations, the Somali population, the Egyptian population- and the Karamoja population from Uganda, was found in the Karamoja (mean ± SE 8.37 ± 0.63) followed by the Zagawa (8.27 ± 0.74), the Nuba (8.04 ± 0.67) and the Nilotic (8.01 ± 0.67) (Figure 2A) populations. The mean number of private alleles was greatest in the Somali (0.400 ± 0.171), followed by the Nilotic (0.292 ± 0.093), the Zagawa (0.273 ± 0.122) and the Karamoja (0.250 ± 0.066) groups (Figure 2B). This observation of greater levels of diversity for the Nilo-Saharan populations from southern and western Sudan and for the Karamoja population from Uganda indicates larger effective population sizes of Nilo-Saharan populations compared with Afro-Asiatic populations. The low number of private alleles found for the Beja (0.031 ± 0.012) and the Nubian (0.129 ± 0.040) (Figure 2B) groups could be the result of high migration rate and greater gene flow from Eurasian populations, because these populations traditionally occupy the entry ports to Sudan, that is, the Beja at Red Sea and the Nubian at the Nile River Valley. A similar result was found previously [4], based on Y-chromosome data. The low number of private alleles in the Coptic groups (0.075 ± 0.053) may be a result of the recent migration of this population from Egypt, where they may have been influenced by gene flow from Asia and Europe. The Nuba also contained few private alleles (0.123 ± 0.065), which may be a consequence of the population being an amalgamation of many groups with high levels of diversity (Hassan *et al*., unpublished data).

**Figure 3:**
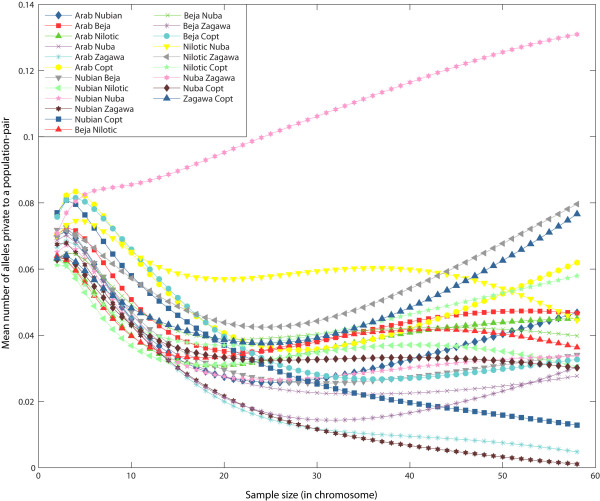
**Mean number of alleles private to a pair of Sudanese populations as a function of the sample size *g *(in chromosomes)**.

**Figure 4:**
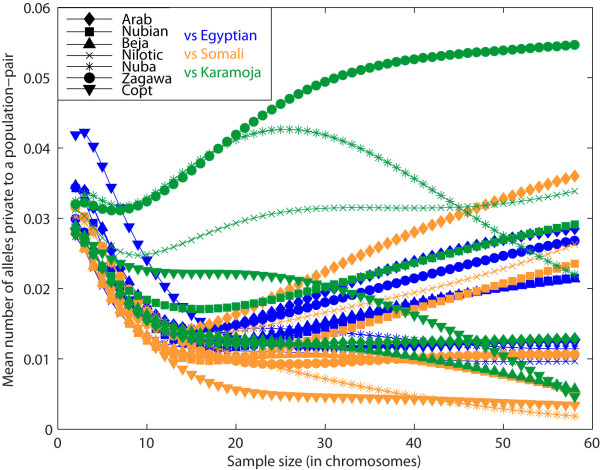
**Mean number of alleles private to a pair of populations as a function of the sample size *g *(in chromosomes)**. Comparisons between each Sudanese population and the Egyptian population is shown in blue; between each Sudanese population and the Somali population in orange; and between each Sudanese population and the Karamoja population from Uganda is shown in green.

For pairs of Sudanese populations, the greatest mean number of alleles that are private to pairs of populations was found for the Zagawa-Nuba pair (0.131 ± 0.064), followed by the Zagawa-Nilotic pair (0.080 ± 0.051) and the Zagawa-Copt pair (0.076 ± 0.063; Figure 3). The lowest number of private alleles for pairs of populations were typically found when we compared a western group (for example, the Zagawa) with a northern (for example, the Nubian) or a central (for example, the Arab) group. When the Sudanese populations were compared with their neighboring populations (sample size of 58 chromosomes), three of the four highest numbers of private alleles for population pairs were seen between the Karamoja population (from Uganda) and either the Zagawa (0.055 ± 0.026), the Nilotic (0.034 ± 0.012) or the Nubian (0.029 ± 0.021) populations (Figure 4), indicating gene flow and/or shared ancestry between the Karamoja population and Nilo-Saharan populations. For smaller sample sizes (10-44 chromosomes), the Zagawa-Nuba pair also has a high number of private alleles. A similar result was found in a previous Y-chromosome study [6], in which all Nilo-Saharan populations (which included the Zagawa, the Nilotic and the Nubian) had little evidence of gene flow with other Sudanese populations. The second largest value for the number of private alleles for population pairs in our study was for the Arab-Somali pair (0.036 ± 0.029; Figure 4), which may be a result of the influence of Arab groups in east Africa as the product of continuous migrations from the Arabian Peninsula across the Gate of Tears over the past three millennia [31]. Among pairs of populations that included the Egyptian population, the Egyptian-Copt pair had the greatest number of private alleles (0.012 ± 0.008) indicating a connection between the Coptic and the Egyptian population (Figure 4).

**Figure 5:**
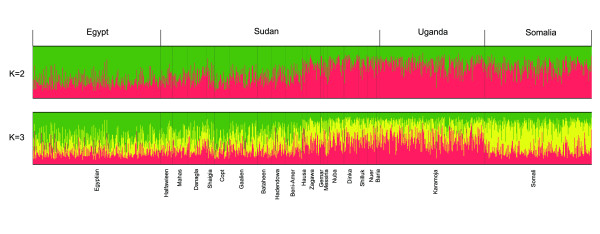
**Clustering of 454 people from 18 Sudanese populations, 218 people from the Karamoja population (Uganda), 265 people from five Egyptian populations and 230 people from one Somali population assuming two or three clusters (*K*)**.The mean (across replicate runs) log likelihood was -64611 for *K *= 2 and -64853 for *K *= 3. Each individual is represented by a column divided into K colors with each color representing a cluster. Different populations are separated by a black line and are labeled below the figure by self-reported ethnicity and above the figure by geographic region.

**Figure 6:**
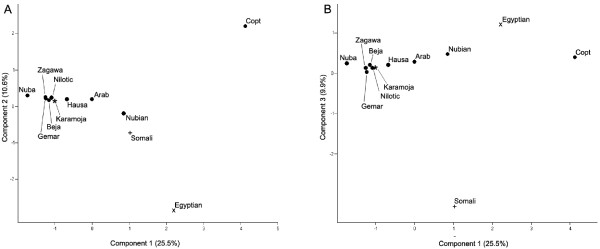
**Principal components based on genetic distance of the Sudanese populations and the Egyptian, the Somali and the Karamoja populations**. The populations that have been genotyped in this study are indicated by filled circles. A) The first and the second principal components. B) The first and the third principal components.

We estimated population structure for the Sudanese populations together with the previously published data from the Somali, Egyptian and Karamoja populations using the software program *Structure *(version 2.3.3; http://pritch.bsd.uchicago.edu/software/structure2_2.html) [32]. Although most individuals had substantial parts of their genomes assigned to more than one cluster, three main patterns could be distinguished (Figure 5). Individuals from northern Sudan and Egypt were (generally) more likely to fall within the 'green' cluster (*P *< 0.001, Mann-Whitney *U*-test), these from southern Sudan and Uganda into the 'red' cluster (*P *< 0.001, Mann-Whitney *U*-test), and those from Somalia into a (partly) separate 'yellow' cluster (*P *< 0.001, Mann-Whitney *U*-test). The mixed ancestry of basically all individuals is probably a consequence of the limited number of markers (and of the set of markers not being particularly informative about ancestry) rather than an indication of recent admixture, a behavior of admixture analyses that has been reported previously (see Figure 4 in Rosenberg *et al. *[29]). Using principal component analysis (PCA), based on pairwise genetic distance between populations, the first principal component explained 25.5% of the genetic variation, and distinguished the Coptic, Egyptian, Somali and Nubian populations from the others (Figure 6A). The second component (10.6%) distinguished the Egyptian, and to some extent, the Somali and the Nubian population from most of the others (Figure 6A), and the third component (9.9%) distinguished the Somali population from all others (Figure 6B). PCA indicated that the Egyptian, Coptic, Somali, and to some extent the Nubian groups form genetically distinct populations. The Nuba, Zagawa, Nilotic and Gemar groups clustered with the Karamoja group from Uganda, as was also indicated by the clustering using *Structure *(Figure 5).

The number of unique alleles (Figure 2B) was greatest in the Somali population, and and in the population structure analyses (Figure 5), the Somali population grouped separately from other populations. Because the Somali population is separated both geographically and linguistically from the other populations included in our study, it is not surprising that it is also genetically distinct. It is possible that the Bantu expansion from West Africa had a stronger effect on the region of the Horn of Africa, where Somalia is located, compared with the region where Sudan is located. For example, the languages in Somalia belong to two major linguistic families, the Afro-Asiatic and Niger-Congo, whereas Nilo-Saharan is absent and the Bantu Swahili language is one of the major languages in Somalia (Ethnologue [1]). Another explanation could be that the Somali population is of both Eurasian and sub-Saharan origin, as suggested by a recent study [33], potentially explaining the differentiation of this population from some east African groups, although many of the Sudanese populations, such as Arabs and the Beja, may also have mixed Eurasian and sub-Saharan origin.

The patterns of population structure we found in northeast Africa, in particular the similarity of Nubian (a northern Sudanese group that speak Nilo-Saharan languages) and the Egyptian population. is consistent with the historical evidence for long-term interactions between Egypt and Nubia, probably resulting in genetic flow between the two regions. However, the Nubian group and the Karamoja group from Uganda share a relatively large number of private alleles (Figure 4), potentially reflecting the shared ancestry of the Nubians with populations from southern Sudan and Uganda. Our results, in addition to mtDNA [7] and Y-chromosome [6,34,35] data, suggest that migration, potentially bidirectional, occurred along the Nile between Egypt and Nubia.

Even though most of the genetic variation was seen within populations and between individuals, some genetic variation was found between groups (Table 4). For the genetic variation between groups, only a fraction of it could be attributed to the linguistic differences between groups (0.21%) or to the geographic distance between the locations of the groups (0.52%). However, at least within Sudan, geography plays a more important role in causing genetic differences between groups compared with the influence of language.

**Table 4:** Partition of genetic variation between the Sudanese populations (using analysis of molecular variance) based on linguistic or geographic classification.

Grouping criteria (*n*)	Groups	Variance (%)		
		
		Within populations	Between populations, within groups	Between groups
Linguistic groups,^a ^(2)	Afro-Asiatic, Nilo-Saharan	99.07	0.73	0.21
Geographic groups, (5)	Central, Northern, Eastern, Western and Southern^b^	98.99	0.49	0.52

For all *F*-statistics, p < 0.01

^a^See Figure 1 and Table 1 for the geographic and linguistic classification of populations.

^b^The Nuba population was excluded because the group included individuals speaking different languages belonging to the Niger-Congo and Nilo-Saharan linguistic families.

## Conclusion

Even though the 15 Identifiler microsatellites are not the most informative markers for inference of ancestry, these markers contain sufficient information to differentiate (to some degree) between distinct geographic and linguistic groups within Sudan and within a larger collection of northeast African populations. We conclude that geography, language and culture have played an important role in shaping patterns and structure of genetic variation in Sudan, and that these patterns may have been shaped by long-term occupation by Nilo-Saharan groups and more recent migration from North Africa and Eurasia to the Nile Valley, but a larger number of markers would be needed for fine-scale population structure inference. This study confirms that the set of 15 Identifiler microsatellites is a suitable tool for personal identification and parentage analysis in Sudan, despite the levels of population structure.

## Methods

The study was described to each participant before sampling, and informed consent was obtained from each participant.

### Samples

In total, 498 unrelated subjects (366 men, 132 women) were recruited from different geographic regions of Sudan. For each individual, we collected blood and (self-reported) information about the gender and ethnic background of the participant and their parents and grandparents. For the calculation of forensic summary statistics, we excluded seven people because of a potential first-degree relationship, which was inferred using Relpair v2.0.1 [36,37], leaving 491 subjects. For the population-genetics analyses, we excluded (i) the same 7 subjects because of a potential first-degree relationship, (ii) 29 for whom the sample size of the particular population was <6, and (iii) eight who failed genotyping for at least one marker, resulting in a final subject group size of 454 from 18 Sudanese populations. The geographic locations of the sampled populations are indicated on a map of Sudan (Figure 1), and sample sizes for the populations are given in Table 1. For the population-genetics analyses, we also combined the genotype data from our Sudanese sample (n = 454) with previously published genotype data from Uganda [25], Egypt [26] and Somalia [27].

### Genetic markers

Genomic DNA was extracted from DNA storage cards (FTA Classic Cards; Whatman, Maidstone, Kent, UK), following the manufacturer's instructions. Fifteen STR loci (D3S1358, vWA, FGA, D8S1179, D21S11, D18S51, D5S818, D13S317, D16S539, TH01, TPOX, CSF1PO, D7S820, D2S1338 and D19S433) were co-amplified for each of the 498 subjects (AmpF*l*STR^® ^Identifiler™ PCR Amplification Kit; Applied Biosystems) following the manufacturer's recommendations [38]. The amplified PCR products were genotyped using an automatic analyzer (ABI PRISM 3730 *XL *Genetic Analyzer; Applied Biosystems). For each run on the analyzer, an allelic ladder, positive and negative controls, and an internal lane standard (GeneScan-600 LIZ; Applied Biosystems) were included. Allele calling was performed (GeneMapper ID software, version 4.0; Applied Biosystems). We followed the International Society for Forensic Genetics (ISFG) recommendations on the analysis of DNA polymorphisms. The recommended nomenclature was used, and we followed the guidelines on quality control [39].

### Analysis of genotype data

Standard summary statistics for forensic applications, including allele frequencies, PE, power of discrimination, and polymorphism information content, were computed (PowerStats, version 12.0; Promega Corp., Madison, WI, USA; http://www.promega.com/geneticidtools/powerstats/). Testing for deviations from Hardy-Weinberg equilibrium was performed using Arlequin, version 3.11; (http://cmpg.unibe.ch/software/arlequin3/) [28]. To assess the power of the 15 Identifiler microsatellite loci for population structure inference, we calculated the informativeness for assignment, *I*_n_, [29] for each locus. The *I*_n _statistic is based on information theory, and evaluates the efficiency of a marker for assigning individuals to one of *K *populations. Following Rosenberg *et al. *[29] for a particular locus,

where *N *is the number of alleles for the locus, *K *is the number of populations, *p_ij_*is the (parametric) frequency of allele *j *in population *i*, *p_j_*is the mean frequency of allele *j *across populations and 'log' denotes the natural logarithm. The minimal *I*_n _value is 0 (when all alleles have equal frequencies in all populations), and the maximal value is log *K *(which occurs when no allele is found in more than one population). *I*_n _was computed for the combined dataset of northeast African populations from Sudan, Egypt, Somalia and Uganda. For comparison of the level of informativeness for the 15 Identifiler microsatellites, we computed *I*_n _values for 377 microsatellites genotyped in the HGDP [29]. Average *I*_n _values were computed for two groups of populations from the HGDP: six African populations (Kenyan Bantu speakers, Mandenka, Yoruba, San, Mbuti Pygmy, Biaka Pygmy), referred to as the 'HGDP sub-Saharan group', and a subgroup of three populations from the HGDP who all speak Niger-Congo languages (Kenyan Bantu speakers, Mandenka, Yoruba), referred to as the 'HGDP Niger-Congo group'.

Based on the genotype data from the 15 microsatellites, we inferred population structure for northeast African populations using the clustering software Structure [32,40]. We used the admixture model, using the F model of correlated allele frequencies across clusters. Each replicate Structure run used a burn-in period of 100,000 iterations, followed by 10,000 iterations from which estimates were obtained. We replicated the Structure analysis 10 times for each choice, based on the number of assumed clusters (*K*), from *K *= 2 to *K *= 10. The 10 replicates for each choice of *K *was summarized (CLUMPP software; http://rosenberglab.bioinformatics.med.umich.edu/clumpp.html[41]), with the Large K Greedy algorithm (10,000 random permutations) to identify common modes of replicates, and to combine the clustering-results across replicates. The combined clustering result was visualized (*distruct *package; http://rosenberglab.bioinformatics.med.umich.edu/distruct.html[42]). Population structure was also assessed through calculating the mean squared distance for microsatellite loci [43] between pairs of populations (*Populations *version 1.2.30; http://bioinformatics.org/project/?group_id = 84). The distance matrix was visualized using PCA.

We computed the mean number of distinct alleles, the mean number of private alleles and the mean number of private alleles for pairs of populations (ADZE; http://rosenberglab.bioinformatics.med.umich.edu/adze.html[44]) for the Sudanese populations (Table 1). For this analysis, we merged populations into larger 'ethnic groups' to avoid small sample sizes, and we also excluded the Gemar because of the small sample size (n = 6), and because they could not be grouped with any of the other populations. This analysis was repeated for the Sudanese ethnic groups, including the populations from Egypt, Somalia and Uganda (the Karamoja). The sample from Egypt was collected from five populations, but because no structure could be detected in these five populations [26], we treated them as one population. Analysis of molecular variance (AMOVA) [45] was used to partition genetic variation for predefined linguistic and geographic groups (Arlequin version 3.11 [28]) and the Nuba population was excluded because the group encompassed individuals speaking different languages belonging to the Niger-Congo and Nilo-Saharan linguistic divisions.

## Competing interests

The authors declare that they have no competing interests.

## Authors' contributions

HB and MJ conceived of the study; HB collected samples with help from HH; HB conducted the genotyping; HB, CS and MJ analyzed the data; and HB, CS and MJ wrote the paper with input from HH. All authors read and approved the final manuscript.
